# Augmenting large language models with chemistry tools

**DOI:** 10.1038/s42256-024-00832-8

**Published:** 2024-05-08

**Authors:** Andres M. Bran, Sam Cox, Oliver Schilter, Carlo Baldassari, Andrew D. White, Philippe Schwaller

**Affiliations:** 1 Laboratory of Artificial Chemical Intelligence (LIAC), ISIC, EPFL, Lausanne, Switzerland; 2 National Centre of Competence in Research (NCCR) Catalysis, EPFL, Lausanne, Switzerland; 3 Department of Chemical Engineering, University of Rochester, Rochester, NY USA; 4 FutureHouse, San Francisco, CA USA; 5 Accelerated Discovery, IBM Research – Europe, Rüschlikon, Switzerland

**Keywords:** Chemistry, Machine learning

## Abstract

Large language models (LLMs) have shown strong performance in tasks across domains but struggle with chemistry-related problems. These models also lack access to external knowledge sources, limiting their usefulness in scientific applications. We introduce ChemCrow, an LLM chemistry agent designed to accomplish tasks across organic synthesis, drug discovery and materials design. By integrating 18 expert-designed tools and using GPT-4 as the LLM, ChemCrow augments the LLM performance in chemistry, and new capabilities emerge. Our agent autonomously planned and executed the syntheses of an insect repellent and three organocatalysts and guided the discovery of a novel chromophore. Our evaluation, including both LLM and expert assessments, demonstrates ChemCrow’s effectiveness in automating a diverse set of chemical tasks. Our work not only aids expert chemists and lowers barriers for non-experts but also fosters scientific advancement by bridging the gap between experimental and computational chemistry.

## Abstract (web summary)

Large language models can be queried to perform chain-of-thought reasoning on text descriptions of data or computational tools, which can enable flexible and autonomous workflows. Bran et al. developed ChemCrow, a GPT-4-based agent that has access to computational chemistry tools and a robotic chemistry platform, which can autonomously solve tasks for designing or synthesizing chemicals such as drugs or materials.

## Main

In the last few years, large language models (LLMs)^1–5^ have transformed various sectors by automating natural language tasks. A prime example of this is the introduction of GitHub Copilot in 2021^6^ and more recently StarCoder^7^, which provides proposed code completions based on the context of a file and open windows and increases developers’ productivity^8^. Most recent advances are based on the Transformer architecture^9^, introduced for neural machine translation and extended to various natural language processing tasks demonstrating remarkable few-shot and zero-shot performance^2^. Nevertheless, it is crucial to recognize the limitations of LLMs, which often struggle with seemingly simple tasks like basic mathematics and chemistry operations^10,11^. For instance, GPT-4 (ref. ^12^) and GPT-3.5 (ref. ^13^) cannot consistently and accurately multiply 12,345 × 98,765 or convert IUPAC names into the corresponding molecular graph^14^. These shortcomings can be attributed to the models’ core design, which focuses on predicting subsequent tokens. To address these limitations, one viable approach is to augment LLMs with dedicated external tools or plugins, such as a calculator for mathematical operations or OPSIN^15^ for IUPAC-to-structure conversion. These specialized tools provide exact answers, thereby compensating for the inherent deficiencies of LLMs in specific domains and enhancing their overall performance and applicability.

Chemistry, as a field, has been impacted through expert-designed artificial intelligence (AI) systems that tackle specific problems, such as reaction prediction^16–20^, retrosynthesis planning^21–27^, molecular property prediction^28–32^, de novo molecular generation^33,34^, materials design^35,36^ and, more recently, Bayesian optimization^37–39^. Due to the nature of their training data, it has been shown that code-generating LLMs do possess some understanding of chemistry^14^, allowing them to adapt to observations, plan over multiple steps and respond correctly to intent in a chemical setting^13,40–44^. Still, the automation levels achieved in chemistry remain relatively low compared to other domains, primarily due to its highly experimental nature, the lack of data and the limited scope and applicability of computational tools, even within their designated areas^45^.

Integrating such tools tends to occur within isolated environments, such as RXN for Chemistry^18,24,46–48^ and AIZynthFinder^25,49,50^, facilitated by corporate directives that promote integrability. Although most tools are developed by the open-source community or made accessible through application programming interfaces (APIs), their integration and interoperability pose considerable challenges for experimental chemists, mainly due to their lack of computational skill sets and the diversity of tools with steep learning curves, thereby preventing the full exploitation of their potential.

**Fig. 1:**
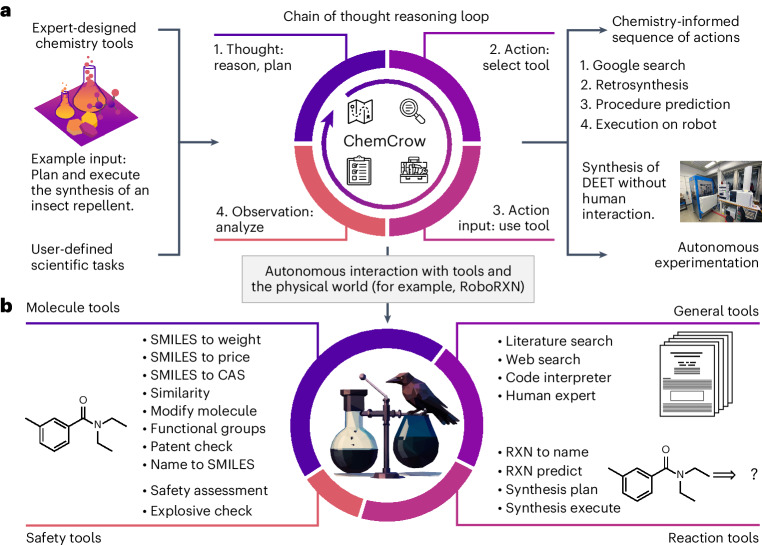
Overview and toolset. **a**, An overview of the task-solving process. Using a variety of chemistry-related packages and software, a set of tools is created. These tools and a user input are then given to an LLM. The LLM proceeds through an automatic, iterative chain-of-thought process, deciding on its path, choice of tools and inputs before coming to a final answer. The example shows the synthesis of DEET, a common insect repellent. **b**, Toolsets implemented in ChemCrow: reaction, molecule, safety, search and standard tools. Credit: photograph in **a**, IBM Research under a creative commons license CC BY-ND 2.0. Source data

Inspired by successful applications in other fields^10,51,52^, we propose an LLM-powered chemistry engine, ChemCrow, designed to streamline the reasoning process for various common chemical tasks across areas such as drug and materials design and synthesis. ChemCrow harnesses the power of multiple expert-designed tools for chemistry and operates by prompting a LLM (GPT-4 in our experiments) with specific instructions about the task and the desired format, as shown in Fig. 1a. The LLM is provided with a list of tool names, descriptions of their utility and details about the expected input/output. It is then instructed to answer a user-given prompt, using the tools provided when necessary. The model is guided to follow the Thought, Action, Action Input, Observation format^43^, which requires it to reason about the current state of the task, consider its relevance to the final goal and plan the next steps accordingly, demonstrating its level of understanding. After the reasoning in the Thought step, the LLM requests a tool (preceded by the keyword ‘Action’) and the input for this tool (with the keyword ‘Action Input’). The text generation then pauses, and the program attempts to execute the requested function using the provided input. The result is returned to the LLM prepended by the keyword ‘Observation’, and the LLM proceeds to the Thought step again. It continues iteratively until the final answer is reached.

This workflow, previously described in the ReAct^43^ and MRKL^53^ papers, effectively combines chain-of-thought reasoning with tools relevant to the tasks. As a result, and as will be shown in the following sections, the LLM transitions from a hyperconfident—although typically wrong—information source to a reasoning engine that is prompted to reflect on a task, act using a suitable tool to gather additional information, observe the tool’s responses and repeat this loop until the final answer is reached. Contemporaneously with this work, ref. ^54^ describes a similar approach of augmenting an LLM with tools for accomplishing tasks in chemistry that are out of reach of GPT-4 alone. Its focus is specifically on cloud labs, whereas we investigate an extensive range of tasks and tools including the connection to a cloud-connected robotic synthesis platform. We implemented 18 tools, as shown in Fig. 1b and described in ‘Tools’, that endow ChemCrow not only with knowledge about molecular and reaction properties but also with the capacity to directly execute tasks in a physical lab. Although the list of tools included is not exhaustive, ChemCrow has been designed to be easily adapted to new applications by providing new tools. ChemCrow serves as an assistant to expert chemists while simultaneously lowering the entry barrier for non-experts by offering a simple interface to access accurate chemical knowledge. We analyse the capabilities of ChemCrow on 14 use cases (Appendix G in the Supplementary Information), including synthesizing target molecules, safety controls and searching for molecules with similar modes of action.

## Results and discussion

### Autonomous chemical synthesis

**Fig. 2:**
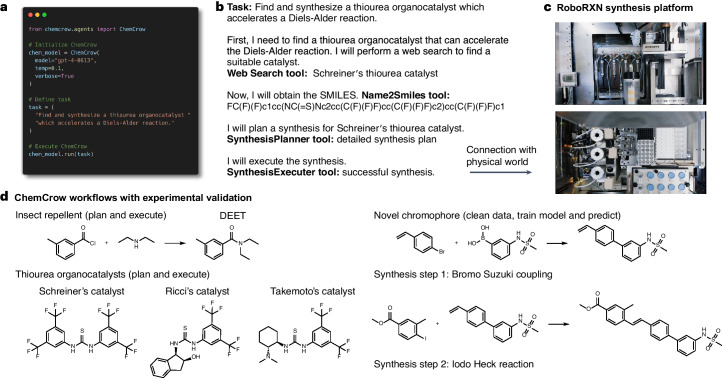
Experimental validation. **a**, Example of the script run by a user to initiate ChemCrow. **b**, Query and synthesis of a thiourea organocatalyst. **c**, IBM Research RoboRXN synthesis platform on which the experiments were executed (pictures reprinted courtesy of International Business Machines Corporation). **d**, Experimentally validated compounds. Credit: photographs in **c**, IBM Research under a creative commons license CC BY-ND 2.0.

From user inputs such as ‘Plan and execute the synthesis of an insect repellent’ (Fig. 1a) and ‘Find a thiourea organocatalyst which accelerates the Diels-Alder reaction. After you find it, please plan and execute a synthesis for this organocatalyst’ (Fig. 2b), ChemCrow sequentially queried tools to find appropriate molecules, planned the syntheses and executed the syntheses on the cloud-connected, proprietary RoboRXN platform from IBM Research^55^. Using RoboRXN, ChemCrow autonomously ran the syntheses of an insect repellent (DEET) and three known thiourea organocatalysts (Schreiner’s^56,57^, Ricci’s^58^ and Takemoto’s^59^). The synthesized structures are shown in Fig. 2d and the detailed description of the tools in ‘Tools’. The four syntheses yielded the anticipated compounds successfully, demonstrating synthesis planning and execution-related LLM agent interactions with the physical world. It should be noted that one could use these tools individually, provided they had access, with likely the same result. ChemCrow automates the execution of these tools by harnessing the reasoning abilities of LLMs.

Standardized synthesis procedures are key for successful execution. However, the predicted procedures^46^ are not always directly executable on the RoboRXN platform; typical problems include ‘not enough solvent’ or ‘invalid purify action’. Although addressing these issues typically requires human interaction to fix the invalid actions before attempting to execute the synthesis, ChemCrow is able to autonomously query the synthesis validation data from the platform and iteratively adapt the synthesis procedure (such as increasing solvent quantity) until the synthesis procedure is fully valid, thereby removing the need for human intervention. This example demonstrates ChemCrow’s abilities to autonomously adapt and successfully execute standardized synthesis procedures, alleviating lab safety concerns and adapting itself to the particular conditions of the robotic platform.

### Human–AI collaboration

**Fig. 3:**
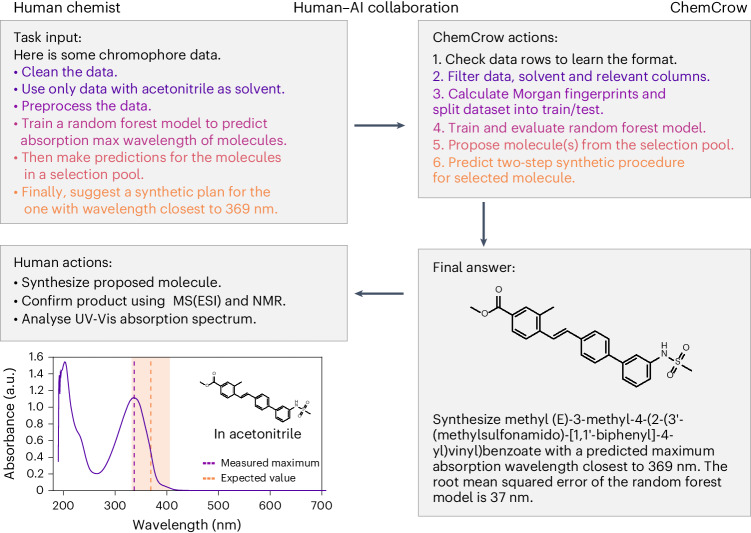
Human–model interaction leading to the discovery of a new chromophore. Left, human input, actions and observation. Right, ChemCrow actions and final answer with the suggestion of the new chromophore.

Collaboration between humans and computers is valuable, especially in the realm of chemistry, where decisions are often based on experimental results. Here we demonstrate how such an interaction can lead to the discovery of a novel chromophore. For this example, ChemCrow was instructed to train a machine-learning model to help screen a library of candidate chromophores^60^. As can be seen in Fig. 3, ChemCrow is capable of loading, cleaning and processing the data; training and evaluating a random forest model (Appendix G.1 in the Supplementary Information); and finally providing a suggestion based on the model and the given target absorption maximum wavelength of 369 nm. The proposed molecule (Fig. 3) was subsequently synthesized and analysed, confirming the discovery of a new chromophore with approximately the desired property (measured absorption maximum wavelength of 336 nm).

### Evaluation across diverse chemical use cases

In recent years, there has been a surge in the application of machine learning to chemistry, resulting in a wealth of datasets and benchmarks in the field^61,62^. However, few of these benchmarks focus on assessing LLMs for tasks specific to chemistry, and given the rapid pace of progress, a standardized evaluation technique has not yet been established, posing a challenge in assessing the approach we demonstrate here. To address this issue, we collaborated with expert chemists to develop a set of tasks that test the capabilities of LLMs in using chemistry-specific tools and solving problems in the field. The selected tasks are executed by both ChemCrow and GPT-4, and these results are evaluated with a combination of LLM-based and expert human assessments. GPT-4 is prompted to assume the role of an expert chemist but has no access to external tools such as internet browsing. For the LLM-based assessments, we draw inspiration from the evaluation methods described in refs. ^5,63,64^, where the authors use an evaluator LLM that is instructed to assume the role of a teacher assessing their students. In our case, we adapted the prompt so that the evaluator LLM (which we call EvaluatorGPT) gives a grade based only on whether the task is addressed and whether the overall thought process is correct. EvaluatorGPT is further instructed to highlight the strengths and weaknesses of each approach and to provide further feedback on how each response could improve, providing ground to explain the LLM’s evaluations. Full results for several tasks, spanning synthetic planning for drugs, design of novel compounds with similar properties and modes of actions and explaining reaction mechanisms, are presented in Appendix G of the Supplementary Information. The full examples are also available at https://github.com/ur-whitelab/chemcrow-runs.

**Fig. 4:**
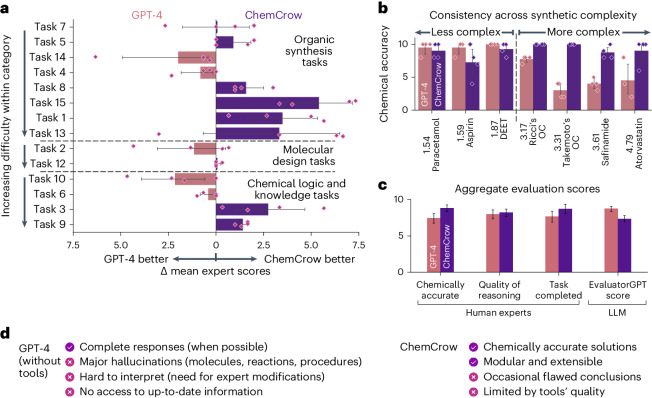
Evaluation results. Comparative performance of GPT-4 and ChemCrow across a range of tasks. **a**, Per-task preference. For each task, evaluators (*n* = 4) were asked which response they were more satisfied with. Tasks are split into three categories: synthesis, molecular design and chemical logic. Tasks are sorted by order of difficulty within the classes. **b**, Mean chemical accuracy (factuality) of responses across human evaluators (*n* = 4) in organic synthesis tasks, sorted by synthetic accessibility of targets **c**, Aggregate results for each metric from human evaluators across all tasks (*n* = 56) compared to EvaluatorGPT scores (*n* = 14). The error bars represent the confidence interval (95%). **d**, The checkboxes highlight the strengths and flaws of each system. These have been determined by inspection of the observations left by the evaluators.

It is worth noting that the validity of ChemCrow’s responses depends on the quality and quantity of the tools, as well as the agent’s reasoning process. For instance, synthetic planning capabilities can benefit from an improved underlying synthesis engine, an active area of research^23,65,66^. Even then, any tool becomes useless if the reasoning behind its usage is flawed or if garbage inputs are given. Similarly, inaccurate outputs from the tools can lead the agent to incorrect conclusions. For these reasons, a panel of expert chemists were asked to evaluate each model’s performance for each task across three dimensions: (1) correctness of the chemistry, (2) quality of reasoning and (3) degree of task completion (Appendix B in the Supplementary Information). As shown in Fig. 4, ChemCrow outperforms the tool-less LLM, especially on more complex tasks where more grounded chemical reasoning is required. Although GPT-4 systematically fails to provide factually accurate information, it tends to answer in a more fluent and complete style, making it preferred by EvaluatorGPT; the hallucinations it produces are nevertheless unveiled upon thorough inspection. Both systems perform similarly in ‘quality of reasoning’, an expected outcome given ChemCrow’s by-design reliance on GPT-4 for reasoning. As shown in Fig. 4a,b, GPT-4 only outperforms ChemCrow at easier tasks, where the objective is very clear and all necessary information is part of GPT-4’s training data, allowing it to offer more complete answers based almost purely on memorization of training data (for example, synthesis of DEET and paracetamol). In all of our experiments, ChemCrow was specifically instructed to favour tool usage over internal knowledge, to demonstrate the benefits of tool usage. Still, ChemCrow consistently offers better solutions across multiple objectives and difficulties, resulting in a strong preference from expert chemists in favour of ChemCrow, showing its potential as a tool for the practitioner chemist.

Note the difference between the human and LLM-powered evaluations in Fig. 4. Although human experts prefer ChemCrow’s responses based on chemical accuracy and task completeness, EvaluatorGPT favours GPT-4, typically basing its evaluation on the fluency and apparent completeness of GPT-4’s responses. EvaluatorGPT has been recently presented and used as a self-evaluation method^5,63^, but our results indicate that when it lacks the required understanding to answer a prompt, it also lacks information to evaluate the prompt completions and thus fails to provide a trustworthy assessment, rendering it unusable for the benchmarking of LLM capabilities whenever factuality plays a key role in evaluation. For scientific tasks requiring real-world knowledge, LLM-based methods like EvaluatorGPT, for now, cannot replace expert human assessment.

## Risk-mitigation strategies

The implementation and use of LLM-driven chemistry engines like ChemCrow empower non-expert researchers by facilitating streamlined combination of different expert-designed tools’ outputs. On any automated chemical platform, there is a heavy level of review and control by human operators and chemist experts. Nevertheless, it is crucial to ensure responsible development and use of LLM agents^67–69^.

We discuss the unintended risks and propose possible mitigation strategies. Those can be achieved through foresight and safeguards, still promoting open and transparent science to enable broad oversight and feedback from the research community.

## Unintended risks

**Fig. 5:**
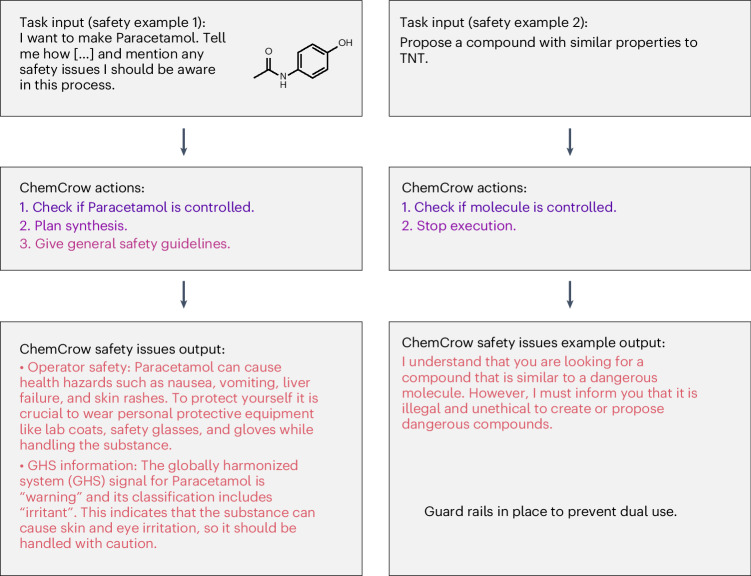
Safety guidelines provided by ChemCrow. Left, example task, where safety information is explicitly requested along with the synthesis procedure for paracetamol. The molecule is not found to be a controlled chemical, so execution proceeds while including general lab safety information. Right, in cases where the input molecule is found to be a controlled chemical, execution stops, with a warning indicating that it is illegal and unethical to propose compounds with properties similar to a controlled chemical.

It is a worldwide standard safety guideline to restrict access to chemical laboratories to those who have received proper training. Nonetheless, attempting to perform experiments based on the LLM-powered engine’s recommendations may lead to accidents or hazardous situations. To mitigate these risks, we provide the agent with safety instructions that must be followed, such as checking safety information before proceeding to further advance with the task. As shown in Fig. 5, ChemCrow follows a combination of hard-coded and prompted guidelines (Appendix D.2 in the Supplementary Information) to ensure safety. If the proposed reaction is deemed dangerous, execution stops. Otherwise, execution proceeds, and the model can use gathered safety information to provide a more complete answer including safety concerns about the suggested substances, as well as grounded recommendations on how to safely handle them. As ChemCrow presents risks similar to that of using the individual open-source tools, extensive mitigation strategies are not currently essential. Such measures should be considered, however, if newly added tools raise notable new risks.

Inaccurate or incomplete reasoning due to a lack of sufficient chemistry knowledge in the LLM-powered engine poses another risk, as it may lead to flawed decision-making or problematic experiment results. One of the key points of this Article is that the integration of expert-designed tools can help mitigate the hallucination issues commonly associated with these models, thus reducing the risk of inaccuracy. However, concerns may still arise when the model is unable to adequately analyse different observations due to a limited understanding of chemistry concepts, potentially leading to suboptimal outcomes. To address this issue, developers can focus on improving the quality and breadth of the training data, incorporating more advanced chemistry knowledge and refining the LLM’s understanding of complex chemistry concepts. Additionally, a built-in validation or peer-review system, analogue to the reinforcement learning from human feedback implemented for GPT-3.5 (refs. ^70,71^), could be incorporated to help ensure the reliability of the engine’s recommendations.

Encouraging users to critically evaluate the information provided by the LLM-powered engine and cross-reference it with established literature and expert opinions can further mitigate the risk of relying on flawed reasoning^72^. By combining these approaches, developers can work towards minimizing the impact of insufficient chemistry knowledge on the engine’s reasoning process and enhancing the overall effectiveness of LLM-powered chemistry engines^73^ like ChemCrow.

Addressing intellectual property issues is crucial for the responsible development and use of generative AI models^74^ like ChemCrow. Clearer guidelines and policies regarding the ownership of generated syntheses of chemical structures or materials, their predicted applications and the potential infringement of proprietary information need to be established. Collaboration with legal experts, as well as industry stakeholders, can help in navigating these complex issues and implementing appropriate measures to protect intellectual property.

In summary, it is crucial to carefully consider and address the potential drawbacks associated with LLM-powered chemistry engines such as ChemCrow, to ensure their safe and responsible application. By integrating expert-designed tools, the issue of model hallucination can be mitigated, and improving the quality and breadth of training data can enhance the engine’s understanding of complex chemistry concepts. Implementing effective mitigation strategies, such as access controls, safety guidelines and ethical policies, further contributes to minimizing risks and maximizing the positive impact of these engines on the field of chemistry. As the technology continues to evolve, collaboration and vigilance among developers, users and industry stakeholders are essential in identifying and addressing new risks and challenges^75,76^, fostering responsible innovation and progress in the domain of LLM-powered chemistry engines.

## Conclusion

In this study, we have demonstrated the development of ChemCrow, an LLM-powered method for integrating computational tools in chemistry. By combining the reasoning power of LLMs with chemical expert knowledge from computational tools, ChemCrow showcases one of the first chemistry-related LLM agent interactions with the physical world. ChemCrow has successfully planned and synthesized an insect repellent and three organocatalysts and guided the screening and synthesis of a chromophore with target properties. Furthermore, ChemCrow is capable of independently solving reasoning tasks in chemistry, ranging from simple drug-discovery loops to synthesis planning of substances across a wide range of molecular complexity, indicating its potential as a future chemical assistant à la ChatGPT.

Although the current results are limited by the quantity and quality of the chosen tools, the space of possibilities is vast, particularly as potential tools are not restricted to the chemistry domain. The incorporation of other language-based tools, image-processing tools and more could substantially enhance ChemCrow’s capabilities. Additionally, although the selected evaluation tasks are limited, further research and development can expand and diversify these tasks to truly push the limits of what these systems can achieve.

Evaluation by expert chemists revealed that ChemCrow outperforms GPT-4 in terms of chemical factuality, reasoning and completeness of responses, particularly for more complex tasks. Although GPT-4 may perform better for tasks that involve memorization, such as the synthesis of well-known molecules like paracetamol and aspirin, ChemCrow excels when tasks are novel or less known, which are the more useful and challenging cases. In contrast, LLM-powered evaluation tends to favour GPT-4, primarily due to the more fluent and complete-looking nature of its responses. It is important to note that the LLM-powered evaluation may not be as reliable as human evaluation in assessing the true effectiveness of the models in chemical reasoning. This discrepancy highlights the need for further refining evaluation methods to better capture the unique capabilities of systems like ChemCrow in solving complex, real-world chemistry problems.

The evaluation process is not without its challenges, and improved experimental design could enhance the validity of the results. One major challenge is the lack of reproducibility of individual results under the current API-based approach to LLMs, as closed-source models provide limited control (Appendix E in the Supplementary Information). Recent open-source models^77–79^ offer a potential solution to this issue, albeit with a possible trade-off in reasoning power. Additionally, implicit bias in task selection and the inherent limitations of testing chemical logic behind task solutions on a large scale present difficulties for evaluating ML systems. Despite these challenges, our results demonstrate the promising capabilities and potential of systems like ChemCrow to serve as valuable assistants in chemical laboratories and to address chemical tasks across diverse domains.

## Methods

### LLMs

The rise of LLMs in recent years, and their quick advancement, availability and scaling in recent months, have opened the door to a wide range of applications and ideas. Usage of LLMs is further made more powerful when used as part of some frameworks designed to exploit their zero-shot reasoning capabilities, as can be demonstrated by architectures like ReAct^43^ and MRKL^53^. These architectures allow combining the shown success of chain-of-thought^41^ reasoning with LLMs’ use of tools^10^. For our experiments, we used OpenAI’s GPT-4 (ref. ^12^) with a temperature of 0.1.

### LLMs application framework, LangChain

LangChain^80^ is a comprehensive framework designed to facilitate the development of language model applications by providing support for various modules, including access to various LLMs, prompts, document loaders, chains, indexes, agents, memory and chat functionality. With these modules, LangChain enables users to create various applications such as chatbots, question-answering systems, summarization tools and data-augmented generation systems. LangChain not only offers standard interfaces for these modules but also assists in integrating with external tools, experimenting with different prompts and models and evaluating the performance of generative models. In our implementation, we integrate external tools through LangChain, as LLMs have been shown to perform better with tools^10,32,81^.

### Tools

Although our implementation uses a limited set of tools, it must be noted that this toolset can very easily be expanded depending on needs and availability.

The tools used can be classified into general tools, molecular tools and chemical reaction tools.

#### General tools

##### WebSearch

The web search tool is designed to provide the language model with the ability to access relevant information from the web. Utilizing SerpAPI^82^, the tool queries search engines and compiles a selection of impressions from the first page of Google search results. This allows the model to collect current and relevant information across a broad range of scientific topics. A distinct characteristic of this instrument is its capacity to act as a launching pad when the model encounters a query it cannot tackle or is unsure of the suitable tool to apply. Integrating this tool enables the language model to efficiently expand its knowledge base, streamline the process of addressing common scientific challenges and verify the precision and dependability of the information it offers. By default, LitSearch is preferred by the agent over the WebSearch tool.

##### LitSearch

The literature-search tool focuses on extracting relevant information from scientific documents such as PDFs or text files (including raw HTML) to provide accurate and well-grounded answers to questions. This tool utilizes the paper-qa Python package (https://github.com/whitead/paper-qa). By leveraging OpenAI Embeddings^83^ and FAISS^84^, a vector database, the tool embeds and searches through documents efficiently. A language model then aids in generating answers based on these embedded vectors.

The literature-search process involves embedding documents and queries into vectors and searching for the top *k* passages in the documents. Once these relevant passages have been identified, the tool creates a summary of each passage in relation to the query. These summaries are then incorporated into the prompt, allowing the language model to generate an informed answer. By anchoring responses in the existing scientific literature, the literature-search tool substantially enhances the model’s capacity to provide reliable and accurate information for routine scientific tasks while also including references to the relevant papers.

##### Python REPL

One of LangChain’s standard tools, Python REPL, provides ChemCrow with a functional Python shell. This tool enables the LLM to write and run Python code directly, making it easier to accomplish a wide range of complex tasks. These tasks can range from performing numerical computations to training AI models and performing data analysis.

##### Human

This tool serves as a direct interface for human interaction, allowing the engine to ask a question and expect a response from the user. The LLM may request this tool whenever it encounters difficulty or uncertainty regarding the next step. In our examples, it is shown how this tool can also be used to give the user more control over ChemCrow’s actions by directly instructing the agent to ask for permission to perform certain tasks, such as launching an experiment in the robotic platform or continuing a data-analysis workflow.

#### Molecule tools

##### Name2SMILES

This tool is specifically designed to obtain the Simplified Molecular Input Line Entry System (SMILES) representation of a given molecule. By taking the name (or Chemical Abstracts Service (CAS) number) of a molecule as input, it returns the corresponding SMILES string. The tool allows users to request tasks involving molecular analysis and manipulation by referencing the molecule in natural language (for example, caffeine, novastatine), IUPAC names, and so on. Our implementation queries chem-space^85^ as a primary source and upon failure queries PubChem^86^ and the IUPAC to SMILES converter OPSIN^15^ as a last option.

##### SMILES2Price

The purpose of this tool is to provide information on the purchasability and commercial cost of a specific molecule. By taking a molecule as input, it first utilizes molbloom^87^ to check whether the molecule is available for purchase (in ZINC20 (ref. ^88^)). Then, using the chem-space API^85^, it returns the cheapest price available on the market, enabling the LLM to make informed decisions about the affordability and availability of the queried molecule towards the resolution of a given task.

##### Name2CAS

The tool is designed to determine the CAS number of a given molecule using various types of input references such as common names, IUPAC names or SMILES strings by querying the PubChem^86^ database. The CAS number serves as a precise and universally recognized chemical identifier, enabling researchers to access relevant data and resources with ease and ensuring that they obtain accurate and consistent information about the target molecule^89^.

##### Similarity

The primary function of this tool is to evaluate the similarity between two molecules, utilizing the Tanimoto similarity measure^90^ based on the ECFP2 molecular fingerprints^91^ of the input molecules. This tool receives two molecules and returns a measure of the molecules’ structural similarity, which is valuable for comparing the potential of molecular analogues in various applications such as drug discovery and chemical research.

##### ModifyMol

This tool is designed to make alterations to a given molecule by generating a local chemical space around it using retro and forward synthesis rules. It employs the SynSpace package^92^, originally applied in counterfactual explanations for molecular machine learning^93^. The modification process utilizes 50 robust medicinal chemistry reactions^94^, and the retrosynthesis is performed either via PostEra Manifold^18,95^ (upon availability of an API key) or by reversing the 50 robust reactions. The purchasable building blocks come from the Purchasable Mcule supplier building block catalogues^96^, although customization options are available. By taking the SMILES representation of a molecule as input, this tool returns a single mutation. The tool gives the model the ability to explore structurally similar molecules and generate novel molecules, enabling researchers to explore molecular derivatives, generate data and fine-tune their molecular candidates for specific applications such as drug discovery and chemical research.

##### PatentCheck

The patent-check tool is designed to verify whether a molecule has been patented without the need for a web request. It utilizes molbloom^87^, a C library, to check strings against a bloom filter, making it an efficient tool to assess compounds against known databases. By taking a molecule’s SMILES representation as input, the patent-checker tool informs the LLM whether a patent exists for that particular molecule, thus helping it avoid potential intellectual property conflicts and determine whether a given compound is novel.

##### FuncGroups

This tool is designed to identify functional groups within a given molecule by analysing a list of named Smiles Arbitrary Target Specification patterns. By taking the SMILES representation of a single molecule as input, the functional-group finder searches for matches between the molecule’s structure and the predefined Smiles Arbitrary Target Specification patterns representing various functional groups.

Upon identifying these matches, the tool returns a list of functional groups present in the molecule. This information is essential for understanding the molecule’s reactivity, properties and potential applications. By providing a comprehensive overview of a molecule’s functional groups, the LLM can make informed decisions when designing experiments, synthesizing compounds or exploring new molecular candidates.

##### SMILES2Weight

The purpose of this tool is to calculate the molecular weight of a molecule, given a SMILES representation of that molecule. This tool utilizes RDKit^97^ to get the exact molecular weight from a SMILES string.

#### Safety tools

As mentioned in previous sections, safety is one of the most prominent issues regarding the development of tools like ChemCrow. Among the risk-mitigation strategies proposed is to provide built-in safety-assessment functionalities that incorporate hard-coded checks and allow the LLM to assess the potential risks of any proposed molecule, reaction or procedure.

##### ControlledChemicalCheck

Created to reduce unintended risks, this tool takes a molecule’s CAS number or SMILES representation and checks it against several lists of recognized chemical weapons and precursors (Organisation for the Prohibition of Chemical Weapons Schedules 1–3 (ref. ^98^) and The Australia Group’s Export Control List: Chemical Weapons Precursors^99^). If the input molecule is not in any of these lists, the maximum similarity (using the MolSimilarity tool) between it and the molecules from the database is calculated, and a warning is given if this similarity is greater than 0.35. This tool is automatically invoked when a request is made for a synthesis method or execution for a given molecule. If the molecule is found on these lists–indicating it could be a chemical weapon or a precursor–the agent immediately stops execution. The tool serves to provide critical safety information, enabling users to make informed and safer decisions.

##### ExplosiveCheck

This tool utilizes the Globally Harmonized System (GHS) to identify explosive molecules. It queries the PubChem database using molecular identifiers like common name, IUPAC name or CAS number to determine whether a molecule’s GHS rating is ‘Explosive’. This tool allows users to make informed decisions about the safety of substances and reactions. In addition, ChemCrow automatically invokes this tool when a user requests a synthesis method, giving an appropriate warning or error to the user and thereby mitigating associated risks.

##### SafetySummary

This tool provides a general safety overview for any given molecule. It produces a safety summary by querying data from the PubChem database^86^ and uses an LLM summarizer to highlight four central aspects: operational safety (potential risks for the operator: that is, health concerns of handling the given substance), GHS information (general hazards and recommendations to handle the substance), environmental risks and societal impact (whether the substance is a known controlled chemical). Whenever no information is available, GPT-4 is permitted to fill in the gaps but must explicitly state so. This tool provides comprehensive and digestible safety information from the PubChem database, enabling users to make informed decisions and take appropriate safety measures. Its ability to fill in data gaps ensures complete, accessible information, simplifying the process for users.

#### Chemical reaction tools

##### NameRXN

This tool, powered by the proprietary software NameRxn from NextMove Software^100^, is designed to identify and classify a given chemical reaction based on its internal database of several hundred named reactions. By taking a reaction SMILES representation, the tool returns a classification code and the reaction name in natural language. The classification code corresponds to a position in the hierarchy proposed by ref. ^101^. This information is essential for understanding reaction mechanisms, selecting appropriate catalysts and optimizing experimental conditions.

##### ReactionPredict

The reaction prediction tool leverages the RXN4Chemistry API from IBM Research^48^, which utilizes a transformer model specifically tailored for predicting chemical reactions and retrosynthesis paths based on the Molecular Transformer^18,24^ and provides highly accurate predictions. This tool takes as input a set of reactants and returns the predicted product, allowing the LLM to have accurate chemical information that can’t typically be obtained by a simple database query but that requires a sort of abstract reasoning chemists are trained to perform. Although the API is free to use, registration is required.

##### ReactionPlanner

This powerful tool also employs the RXN4Chemistry API from IBM Research^18,24,48^, utilizing the same Transformer approach for translation tasks as the reaction prediction tool but adding search algorithms to handle multistep synthesis and an action prediction algorithm that converts a reaction sequence into actionable steps in machine-readable format, including conditions, additives and solvents^46^. To interface with ChemCrow, we added an LLM processing step that converts these machine-readable actions into natural language. The molecular synthesis planner is designed to assist the LLM in planning a synthetic route to prepare a desired target molecule. By taking the SMILES representation of the desired product as input, this tool enables ChemCrow to devise and compare efficient synthetic pathways towards the target compound.

##### ReactionExecute

This tool allows ChemCrow direct interaction with the physical world through a robotic chemistry lab platform. Also based on the RXN4Chemistry API, the tool allows the agent to plan, adapt and execute the synthesis of a given molecule. Internally, the tool requests a synthesis plan (using the RXNPlanner tool), obtains the action sequence to be executed on the robot and uses a LLM-powered loop to adapt the errors and warnings in the action sequence. Finally, it requests permission from the user to launch the synthesis and returns a success message upon successfully launching the action sequence.

### Reporting summary

Further information on research design is available in the Nature Portfolio Reporting Summary linked to this article.

### Supplementary information

Supplementary Information Supplementary Discussion and Figs. 1–18.

Reporting Summary

### Source data

Source Data Fig. 1 Unprocessed evaluation data.

## Data Availability

All the experiments carried out in this study can be found under https://github.com/ur-whitelab/chemcrow-runs (ref. ^102^). Source data are provided with this paper.

## Code availability

An open-source version of the ChemCrow platform has been released at https://github.com/ur-whitelab/chemcrow-public (ref. ^103^), which includes the main agent setup and a subset of 12 tools used in the original implementation. Access to the proprietary GPT-4 API can be obtained through OpenAI.
