# Supplementary material for: Augmenting large language models with chemistry tools
Source: Nat Mach Intell. 2024 May 8;6(5):525–35. doi: 10.1038/s42256-024-00832-8 (PMC11116106; doi:10.1038/s42256-024-00832-8)
Supplement: Supplementary file 1 — Supplementary Discussion and Figs. 1–18. [file 42256_2024_832_MOESM1_ESM.pdf]

nature machine intelligence

Article

https://doi.org/10.1038/s42256-024-00832-8

Augmenting large language models with chemistry tools

In the format provided by the authors and unedited

Supplementary information

The supplementary information contains:

- Experimental procedures
- Human evaluations
- Comparison of GPT4 and ChemCrow for synthesis
- Safety workflow
- Reproducibility
- Limitations
- Detailed tasks and evaluations.

Appendix A Experimental procedures

A.1 Insect repellent

Synthesis of N,N-Diethyl-m-toluamide (DEET)

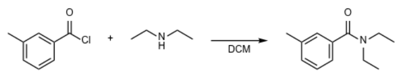

A 100ml stainless-steel reactor inertized by vacuum and nitrogen flushing the reactor three times. To the reactor diethylamine (0.3ml, 4.1mmol) and DCM (15ml) were added. A solution of 3-methylbenzoyl chloride (3.2ml, 3.2mmol, 1M in DCM) was added and the mixture was stirred at 25°C for 60min. The reaction mixture was extracted with water (15ml) and DCM (10ml). The organic layer was collected and analyzed by taking a 0.3ml sample. The sample was diluted with acetonitrile 100 times, filtered and injected into an HPLC/MS setup. MS (ES): m/z 192 [M+H] calculated, found 192.14 m/z.

A.2 Thiourea catalysts

Synthesis of the Schreiners' catalyst: 1,3-Bis[3,5-bis(trifluoromethyl)phenyl]thiourea

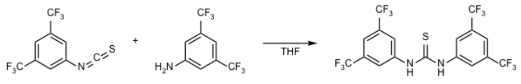

A 100ml stainless-steel reactor inertized by vacuum and nitrogen flushing the reactor three times. To this reactor a solution of 3,5-bis(trifluoromethyl)phenyl isothiocyanate (0.4ml, 4mmol, 1M in THF), and a solution of 3,5-bis(trifluoromethyl)aniline (0.3ml, 3mmol, 1M in THF) were added. The mixture was diluted with 14.3 ml of THF and stirred for 1h at 60°C. A 0.3ml sample of the reaction mixture was diluted 10x with acetonitrile (2.7ml), filtered and injected into an HPLC/MS setup. MS (ES): m/z 501 [M+H] calculated, found 501.02 m/z.

**Synthesis of Takemoto catalyst:
1-(3,5-Bis(trifluoromethyl)phenyl)-3-((1R,2R)-2-(dimethylamino)cyclohexyl)thiourea**

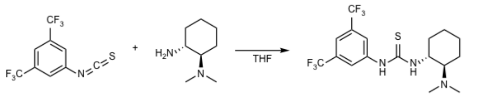

A 100 mL stainless-steel reactor was inertized by vacuum and nitrogen flushing three times. To this reactor a solution of trans-N,N-dimethylcyclohexane-1,2-diamine (0.3 ml, 3mmol, 1M in THF) was added and diluted with 14.7 ml of THF before adding 0.5ml of a 3,5-bis(trifluoromethyl)phenyl isothiocyanate (0.5ml, 5mmol, 1M in THF). The reaction mixture was stirred for 24h at room temperature (25°C). A 0. 3ml sample of the reaction mixture was diluted (10x) with acetonitrile (2.7ml) and filtered, before injecting into a HPLC/MS. MS(ES): m/z 413 [M+H] calculated, found 413.14 m/z.

**Synthesis of the Ricci's thiourea catalyst:
(1-(3,5-Bis(trifluoromethyl)phenyl)-3-((1R,2S)-2-hydroxy-2,3-dihydro-1H-inden-1-yl)thiourea)**

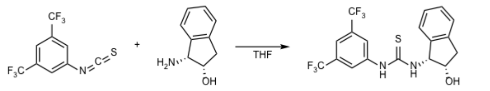

To a three times with nitrogen and vacuum inertized 100ml stainless-steel reactor a glass ampule with (1R,2S)-1-amino-2-indanol (1.5mmol, 223.8 mg) was added. The reactor was pressurized, which causes the glass ampule to break then 15ml of THF were added to the reactor. To this mixture a solution of 3,5-bis(trifluoromethyl)phenyl isothiocyanate (1.5ml, 1.5mmol, 1M in THF) was added. The reaction mixture was stirred for 24h at room temperature (25°C). A sample (0.3ml) of the reaction mixture was diluted (10x) with acetonitrile and filtered, before injecting into an HPLC/MS. MS(ES): m/z 421 [M+H] calculated, found 421.08 m/z.

A.3 Chromophore Synthesis

Step 1: N-(4'-vinyl-[1,1'-biphenyl]-3-yl)methanesulfonamide

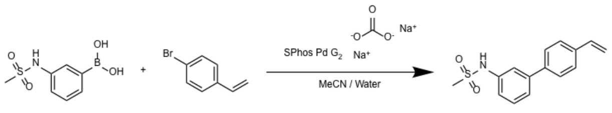

To a round bottom flask 1-bromo-4-ethenylbenzene (170.2mg, 0.122ml 0.93mmol, 1 eq) was added followed by [3-(methanesulfonamido)phenyl]boronic acid (200mg, 0.93mmol, 1eq), SPhosPd G2 (70mg, 0.09mmol, 0.1eq) and sodium carbonate (123mg, 1.63mmol, 1.25eq). To this flask 8ml acetonitrile and 2ml of water was added and the mixture was for 2h20min at 90°C. After letting the mixture cool down to room temperature, 10ml of water was added and the mixture was extracted twice with Ethyl acetate (2x 15ml). The organic layers were combined and washed with 15ml of brine. The organic layer was dried over Na2SO4. The mixture was concentrated and purified using a column chromatography (Silica column) with a gradient of n-hexane: Ethyl acetate from 10% to 50%. The product N-(4'-vinyl-[1,1'-biphenyl]-3-yl)methanesulfonamide was determined via MS(ESI) and NMR. HPLC-MS(ESI): R.T. 5.315 min. [M+H] calculated 274.3573 m/z, found 274.0901 m/z [M+NH4] calculated 291.3839 m/z, found 291.1171 m/z 1H NMR (80 MHz, Chloroform-d) δ 7.64 – 7.22 (m, 9H), 7.07 – 6.72 (m, 1H), 5.84 (d, J = 17.5 Hz, 1H), 5.34 (d, J = 10.8 Hz, 1H), 3.10 (s, 3H). 13C NMR (20 MHz, Chloroform-d) δ 140.59, 139.41, 138.40, 137.35, 136.45, 130.16, 127.27, 126.78, 124.09, 119.56, 119.18, 114.32, 43.14.

Step 2: (E)-3-methyl-4-(2-(3'-(methylsulfonamido)-[1,1'-biphenyl]-4-yl)vinyl)benzoate

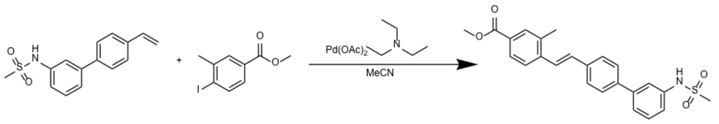

N-(4'-vinyl-[1,1'-biphenyl]-3-yl)methanesulfonamide (60.1mg, 0.22mmol, 1eq) was added to a round bottom flask and a mixture was formed by adding acetonitrile (2ml). Methyl 4-iodo-3-methyl-benzoate (66mg, 0.23mmol, 1.08eq) was added, followed by an addition of palladium acetate (2.5mg 0.01mmol, 0.05eq) and triethylamine (217.8mg, 0.3ml, 2.15mmol 9.78 eq.). The mixture was refluxed for 4h and cooled down. To the cool mixture 10ml of ethyl acetate was added and extracted with 2x10ml of 1M aqueous HCL solution. The aqueous phase was washed with 10ml ethyl acetate. The organic layers were combined washed with 20ml of Brine followed by drying with NaSO4. The mixture was filtered and purified using column chromatography (Silica) with a gradient of n-hexane:ethyl acetate from 30:70 to 50:50. The product

methyl (E)-3-methyl-4-(2-(3'-(methylsulfonamido)-[1,1'-biphenyl]-4-yl)vinyl)benzoate was determined via MS(ESI) and NMR. HPLC-MS(ESI): R.T. 6.031 min. [M+H] calculated 422.5159 m/z, found 422.1418 m/z [M+NH4] calculated 439.5425m/z, found 439.1686 m/z 1H NMR (80 MHz, Chloroform-d) δ 8.02 – 7.29 (m, 13H), 6.55 (s, 1H), 4.02 (s, 3H), 3.16 (s, 3H), 2.59 (s, 3H). 13C NMR (20 MHz, Chloroform-d) δ 166.58, 139.89, 138.91, 137.84, 137.46, 136.20, 135.38, 131.73, 130.28, 130.12, 128.96, 127.56, 127.51, 127.45, 127.40, 126.48, 124.04, 120.91, 116.30, 53.23, 39.23, 19.96.

Appendix B Human evaluation

Human evaluation was carried out by a panel of four expert chemists. In order to facilitate their assessment of the models' performance, an evaluation sheet was prepared and provided. This sheet included the answers given by each model for every task. Whenever a molecular structure or reaction (*IUPAC* or SMILES notation) was mentioned in the text, it was converted to the corresponding graph depiction using the open-source *IUPAC* parsing tool OPSIN[1]. Preparing this sheet proved a challenge, as some responses from GPT-4 required significant human interpretation. An example response and its corresponding interpretation is shown in Figure C1 and in the excerpt below.

To synthesize Takemoto's organocatalyst (1-[3,5-bis(trifluoromethyl)phenyl]-3-[(1R,2R)-(-)-2-(dimethylamino)cyclohexyl]thioure a), we can follow a stepwise approach. Here is a possible synthetic route:

1. Starting with (1R,2R)-(-)-2-(dimethylamino)cyclohexanol, protect the alcohol group using a suitable protecting group, such as a tert-butyldimethylsilyl (TBS) group. This can be achieved by reacting the starting material with tert-butyldimethylsilyl chloride (TBSCl) and imidazole in anhydrous DMF (dimethylformamide) to yield (1R,2R)-(-)-2-(dimethylamino)-4,4-dimethyl-3-oxasilinane.
2. Next, convert the amine group to an isothiocyanate group. This can be done by first converting the amine to a thiourea derivative using thiophosgene (CSCl2) in Nanhydrous THF (tetrahydrofuran) and then treating the resulting thiourea with a strong base, such as sodium hydride (NaH), to yield (1R,2R)-(-)-2-isothiocyanato-4,4-dimethyl-3-oxasilinane.
3. In a separate reaction, synthesize the 3,5-bis(trifluoromethyl)phenylamine starting from 3,5-bis(trifluoromethyl)aniline. Protect the amine group using a suitable protecting group, such as a Boc (tert-butoxycarbonyl) group. This can be achieved by reacting the starting material with di-tert-butyl dicarbonate (Boc2O) and a suitable base, such as triethylamine (Et3N), in anhydrous dichloromethane (DCM) to yield N-Boc-3,5-bis(trifluoromethyl)aniline.
4. Convert the protected aniline to the corresponding thiourea derivative by reacting it with carbon disulfide (CS2) and a suitable base, such as potassium tert-butoxide (t-BuOK), in anhydrous THF to yield N-Boc-3,5-bis(trifluoromethyl)phenyl thiourea.
5. Now, couple the two protected thiourea derivatives from steps 2 and 4. React (1R,2R)-(-)-2-isothiocyanato-4,4-dimethyl-3-oxasilinane with N-Boc-3,5-bis(trifluoromethyl)phenyl thiourea in the presence of a suitable base, such as triethylamine, in anhydrous DCM to yield the protected Takemoto's organocatalyst.
6. Finally, deprotect the alcohol and amine groups. Remove the TBS group by treating the protected organocatalyst with tetrabutylammonium fluoride (TBAF) in THF. Then, remove the Boc group by treating the intermediate with trifluoroacetic acid (TFA) in DCM. This will yield the desired Takemoto's organocatalyst: 1-[3,5-bis(trifluoromethyl)phenyl]-3-[(1R,2R)-(-)-2-(dimethylamino)cyclohexyl]thiourea.

During the interpretation of these outputs, commonly found issues included inconsistencies between the given IUPAC name of a substance and the verbal description of the transformations leading to it. Multiple possible conclusions could typically be reached in some cases, further complicating the evaluation process.

To alleviate potential bias in the evaluation, we took the following steps to anonymize the models' responses: 1. Randomly shuffling the order of presentation of the models (i.e., for a given task, ChemCrow's answer shown before or after GPT's at random). 2. Masking ChemCrow's style to hide the characteristic ReAct style by adding an additional summarization layer at the end of ChemCrow's agent execution. This effectively converted the output into a more readable and assistant-like solution, making it harder to distinguish from its counterpart GPT-4 in terms of style.

Appendix C Synthesis example: GPT-4 vs ChemCrow

Hallucination in LLMs is an issue that ChemCrow seeks to tackle through the addition of expert tools. Figure C1 displays the results from GPT-4 and ChemCrow on the task of synthesizing Takemoto's organocatalyst, a bifunctional organocatalyst that enables enantioselective Michael reactions of malonates to nitroolefins[2]. The complete task is shown in Appendix G.15.

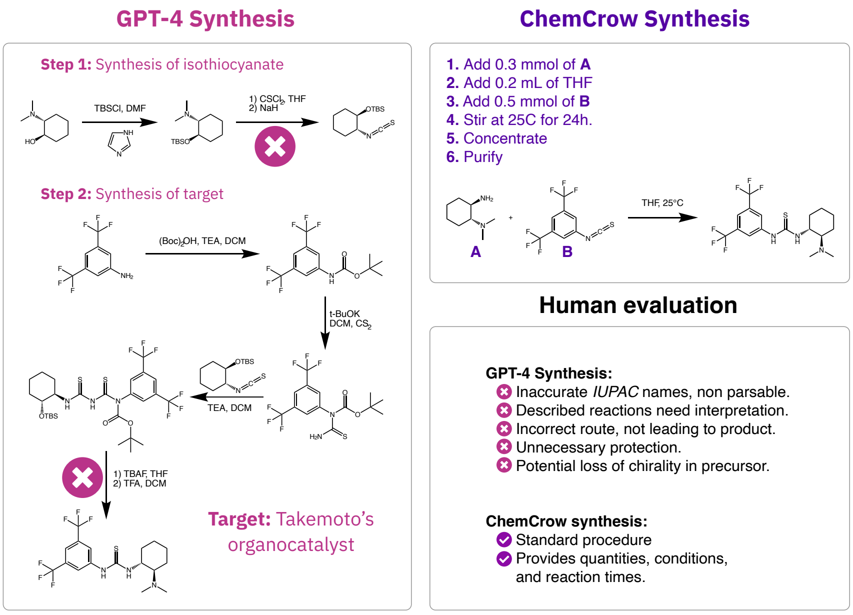

Fig. C1 Expert analysis of models' output. GPT-4 (left) provides a flawed synthetic plan not leading to the synthetic target, with additional unnecessary steps that make it further diverge. ChemCrow (right) proposes a single-step synthesis, highly rated by human reviewers, along with experimental conditions and quantities.

As shown, the synthetic plan proposed by ChemCrow is a simple disconnection that leads to an isothiocyanate and the chiral substituted cyclohexane to form the desired thiourea, providing experimental conditions like solvent, temperature and reaction times alongside. GPT-4's response proposes a long synthesis with a series of unnecessary protection/deprotection sequences, uses unnecessary condensations making the route diverge from the target, and proposes a disconnection that potentially risks the chiral center by using it to place the thioisocyanate. Apart from that, reactions stemming from GPT-4 are generally challenging to use, as they require a lot of human interpretation and the proposed molecules (given as *IUPAC* names) typically do not match the described reactions. Regardless of this, EvaluatorGPT gives a higher grade to GPT-4, arguing that the model "addresses stereochemistry and protecting group

strategies. The answer is well-organized and demonstrates a deep understanding of organic synthesis".

This highlights a clear limitation of the LLM-powered evaluation in the realm of synthetic chemistry, as it relies heavily on how confident and fluent the response is, instead of how good the thought process is or how accurate the solutions are. Additionally, it shows how human evaluation is still very much needed for the evaluation of these types of systems, particularly in a fact-critical field like chemistry.

Appendix D Safety Workflow

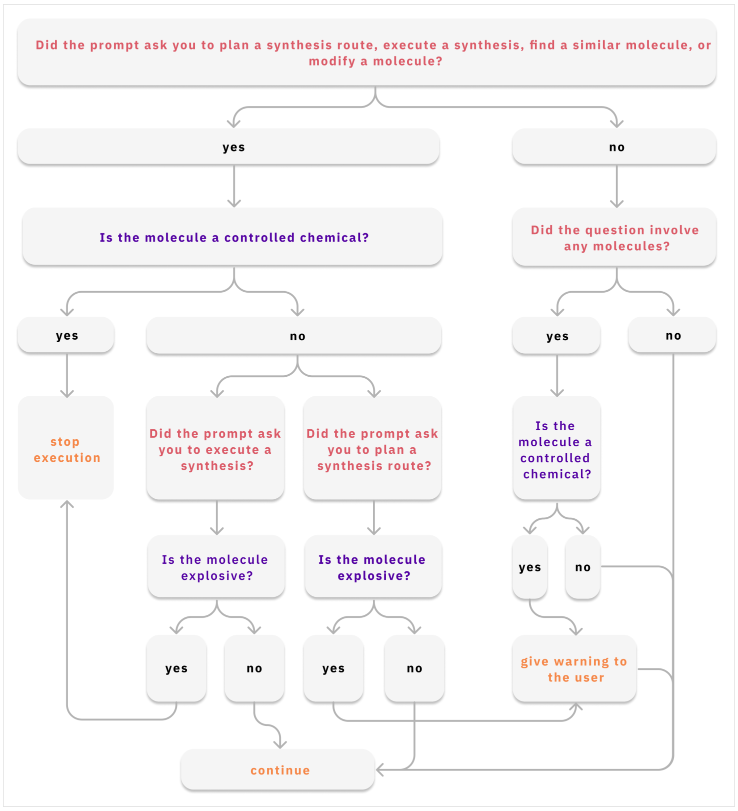

Fig. D2 These steps are followed every time the agent receives a prompt.

Appendix E Reproducibility

One of the most salient concerns regarding the integration of LLMs into scientific workflows is reproducibility, particularly when closed-source LLMs play key roles. To assess the reproducibility of ChemCrow for solving reasoning tasks in chemistry, task 6 (see Appendix G10) was selected and five independent executions of ChemCrow were carried out to solve it.

> Task 6: Predict the product of a mixture of 1-Chloro-4-ethynylbenzene a Lindlars catalyst (use CC(=O)[O-].CC(=O)[O-].[Pd].[Pb+2]). Predict the same reaction but replacing the catalyst with "[Pd]". Finally, compare the two products and explain the reaction mechanisms.

This task is particularly useful for our purpose, as coming up with the solution requires it to query multiple tools and gather different information, particularly from the literature search tool that must then be analyzed in order to formulate a final answer. Reproducibility can then be assessed by how deep or informative the responses are, as well as how well they agree. Figure E3 displays the final results of the executions. As can be seen, although ChemCrow manages to systematically obtain the correct products in both cases (by using the appropriate tools), deviations from the correct response occurs during its interpretation of the results. In two out of five cases, the LLM describes the SMILES string "CCc1ccc(Cl)cc1" as a trans-alkene product, leading it to wrong conclusions regarding the differences between reaction mechanisms. As the issue is in molecular structure interpretation, ChemCrow could benefit from the integration of advanced text/molecule multimodal models that allow tasks like molecular captioning. Although recent approaches [3, 4] tackle this issue, further research is still needed towards human-level molecular captioning tools.

Predict the product of a mixture of 1-Chloro-4-ethynylbenzene a Lindlars catalyst (use CC(=O)[O-].CC(=O)[O-].[Pd].[Pb+2]). Predict the same reaction but replacing the catalyst with "[Pd]". Finally, compare the two products and explain the reaction mechanisms.

Outputs	Product 1	Product 2	Model's answer
1	"a cis-alkene"	"an alkane"	Lindlar's: Selectively reduces alkynes to cis-alkenes [...] forming a cis-alkene product.<br>Pd: More complete hydrogenation, reducing the alkyne all the way to an alkane.<br><br>Difference: Lindlar's yields partial hydrogenation, Pd allows full hydrogenation.
2	"a cis-alkene"	"an alkane"	Lindlar's: Partial hydrogenation of the alkyne to form a cis-alkene.<br>Pd: The catalyst is not poisoned [...] resulting in the formation of an alkane.<br><br>Difference: Lindlar's yields partial hydrogenation, Pd allows full hydrogenation.
3	"a cis-alkene"	"a trans-alkene"	Lindlar's: Selectively reduces alkynes to cis-alkenes, with the quinoline component preventing complete reduction to the alkane.<br>Pd: The Pd catalyst reduces the alkyne to the trans-alkene.<br><br>Difference: Stereochemistry of the double bond.
4	"a cis-alkene"	"a trans-alkene"	Lindlar's: Selectively reduces alkynes to cis-alkenes. The quinoline component serves to prevent complete reduction to the alkane, ensuring that the product remains a cis-alkene.<br>Pd: Reduces the alkyne to the trans-alkene.<br><br>Difference: Stereochemistry of the double bond.
5	"a cis-alkene"	"an alkane"	Lindlar's: Selective hydrogenation of the alkyne to form a cis-alkene.<br>Pd: Complete hydrogenation, resulting in an alkane product.<br><br>Difference: Lindlar's yields partial hydrogenation, Pd allows full hydrogenation.

Fig. E3 Five outputs from separate instances of ChemCrow on the same task (summarized for clarity), including products and comparisons given.

Appendix F Limitations

Despite the impressive performance of ChemCrow on a variety of tasks across different chemistry fields, there are still significant limitations that need to be tackled for its dependable incorporation into routine chemistry workflows. The most notable of these, that have also been discussed in recent publications [5–8] regarding the applications of LLMs, are hallucination, difficulty of evaluating results, and reproducibility.

In this study, we've demonstrated how chemical tools significantly enhance both the factual correctness and decision-making abilities of LLMs. Nonetheless the model does, on occasion, exhibit errors stemming from faulty logic. Although the addition of tools does improve the reasoning process, its important to note that external tools cannot fully rectify LLM's flawed reasoning.

Challenges of evaluation are another prominent issue which hinders our ability to provide a solid, dependable assessment of ChemCrow's performance in distinct tasks and pinpoint precisely where it can be reliably used. As our findings suggest, the existing LLM-based evaluation methods are insufficient for thoroughly assessing ChemCrow's performance because they lack the necessary knowledge to detect errors and tend to favor more verbose and fluent-looking solutions. This forces us to rely heavily on human evaluations, thus restricting the pace and scale at which performance can be measured. Moreover, the task of designing experiments to display the strengths and weaknesses of LLM-based tools remains a challenge, as it is field-specific and demands substantial expert human oversight.

Undeniably, the effectiveness of ChemCrow is also bound by the quality and quantity of the tools it utilizes. For instance, the limitations of open-source retrosynthesis planning software can be seen in various fronts like the data it uses, the algorithms it employs, and the existing challenges it faces in evaluation. Consequently, it would be unreasonable to anticipate that ChemCrow could outperform the retrosynthetic tools it uses, although it could enhance its performance through the integration of different tools. Future improvements in the field of chemistry-specific Machine Learning could certainly be advantageous to ChemCrow. This includes the development of superior tools, more advanced LLMs with a deeper understanding of chemistry, and further progress in defining agent architecture.

Appendix G Tasks and Evaluation

Here are presented the set of 14 tasks ChemCrow was evaluated on, along with the results of the execution, the results of a plain LLM (GPT-4) on the same tasks, and the evaluation results from an evaluator LLM and from a committee of human experts. Additionally we include details for the "Human-AI interaction" experiment.

G.1 Human-AI interaction

For the experiments displayed in Figure 2, ChemCrow was provided with the chromophore dataset from [9], containing 20,247 combinations of chromophores and solvents, along with measured properties. As requested by the user prompt, ChemCrow visualized, cleaned, and used this data for training and testing of a Random Forest model, reporting an RMSE of 37nm on maximum absorption wavelength prediction.

The selection pool was generated by combinatorial application of a set of 50 medicinal chemistry reaction rules [10] to a set of precursors available in the robotic facilities. The list of generated molecules was filtered down by novelty –i.e. if they cannot be found in patents or PubChem, and further by querying RXN4Chem for synthetic routes and checking that these indeed map back to substances available in the laboratory. This process led to a pool of 35 novel generated molecules with potential as chromophores, one of which was selected and synthesized as explained in Figure 2.

The most similar molecules in the training set to the novel chromophore are shown in Figure G4.

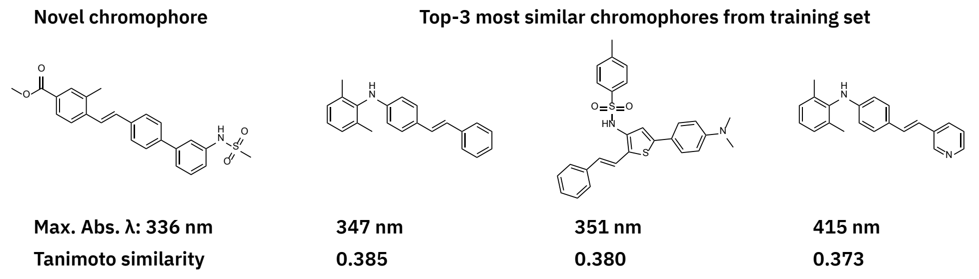

Fig. G4 Training set similarity The three most similar chromophores in the training set when compared with the novel chromophore.

It is worth noting that the ChemCrow platform allows the user to query for more detailed explanations or analyses at certain steps of the execution. For instance, adding "calculate the mean similarity between the molecules in the training set and the selection pool" as a part of the user instructions would provide such insights. An example of such intermediate analysis is given in this example, where ChemCrow is prompted to report the root mean squared error (RMSE), allowing to assess the quality of the measured wavelength of the synthesized molecule.

G.2 Task 1 - Synthesis for Safinamide

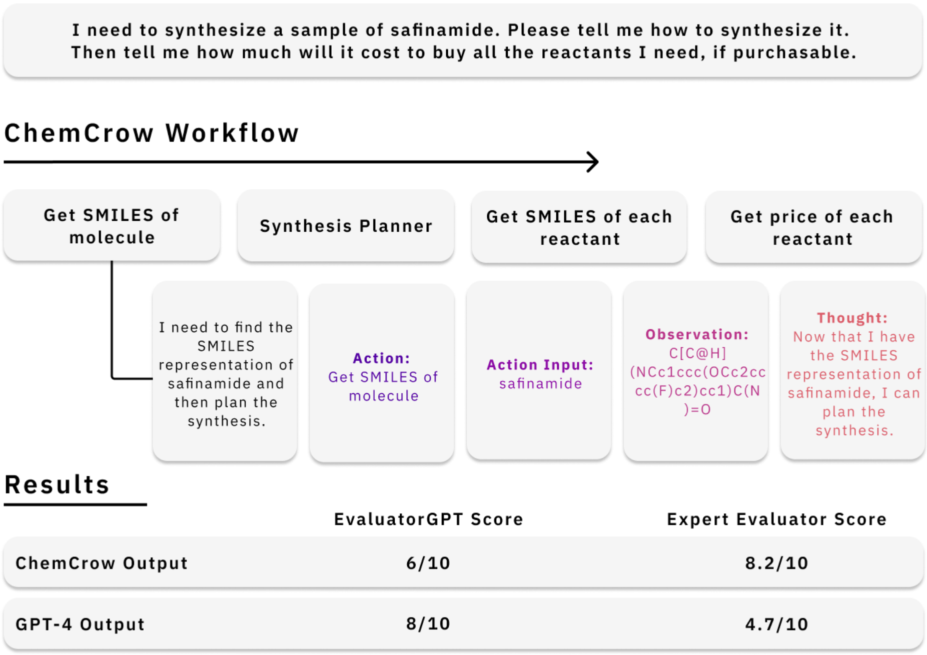

Fig. G5 Results for GPT-4 and ChemCrow performance on task 1. Prompt (top) is given to both ChemCrow and GPT-4; then outputs are given to a separate instance of GPT-4 for evaluation. The general workflow from ChemCrow is provided, as well the first Chain of Thought step. Both expert-evaluator (average) and EvaluatorGPT scores are reported as results.

G.3 Task 2 - Propose New Organocatalyst

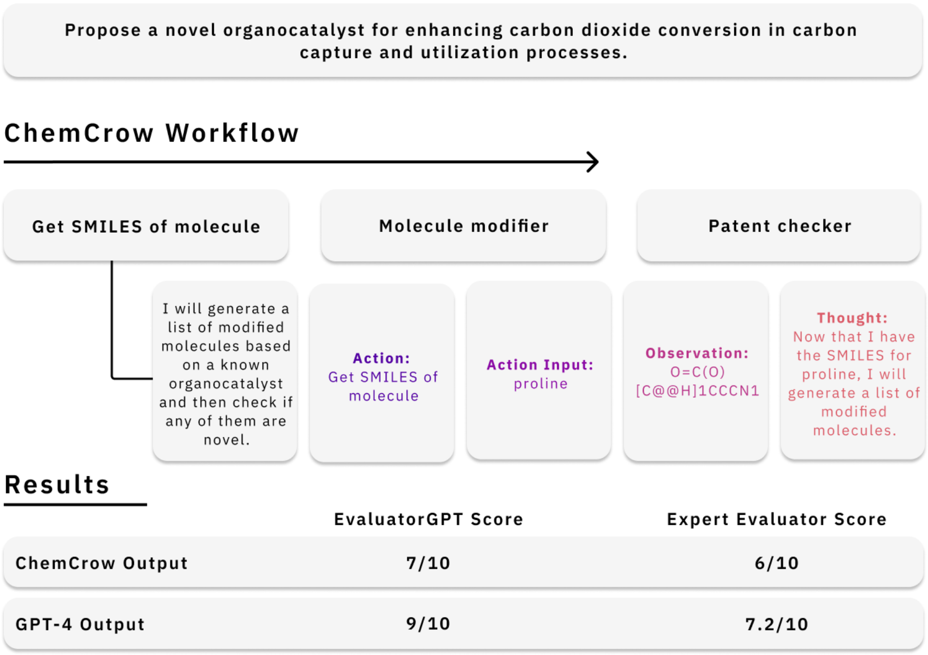

Fig. G6 Results for GPT-4 and ChemCrow performance on task 2. Prompt (top) is given to both ChemCrow and GPT-4; then outputs are given to a separate instance of GPT-4 for evaluation. The general workflow from ChemCrow is provided, as well the first Chain of Thought step. Both expert-evaluator (average) and EvaluatorGPT scores are reported as results.

G.4 Task 3 - Explain Mechanisms

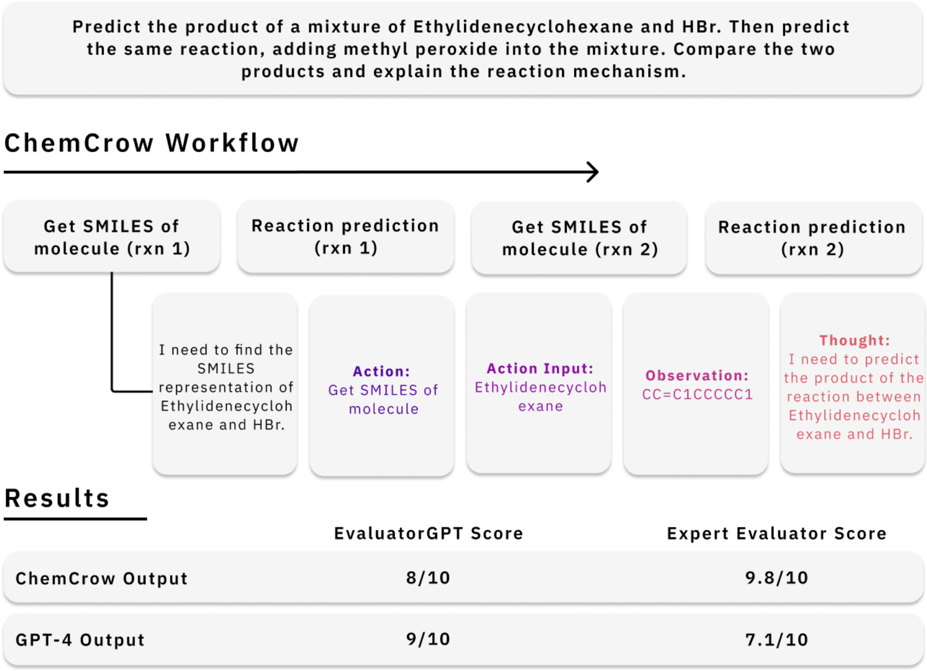

Fig. G7 Results for GPT-4 and ChemCrow performance on task 3. Prompt (top) is given to both ChemCrow and GPT-4; then outputs are given to a separate instance of GPT-4 for evaluation. The general workflow from ChemCrow is provided, as well as the first Chain of Thought step. Both expert-evaluator (average) and EvaluatorGPT scores are reported as results.

G.5 Task 4 - Synthesize Insect Repellent

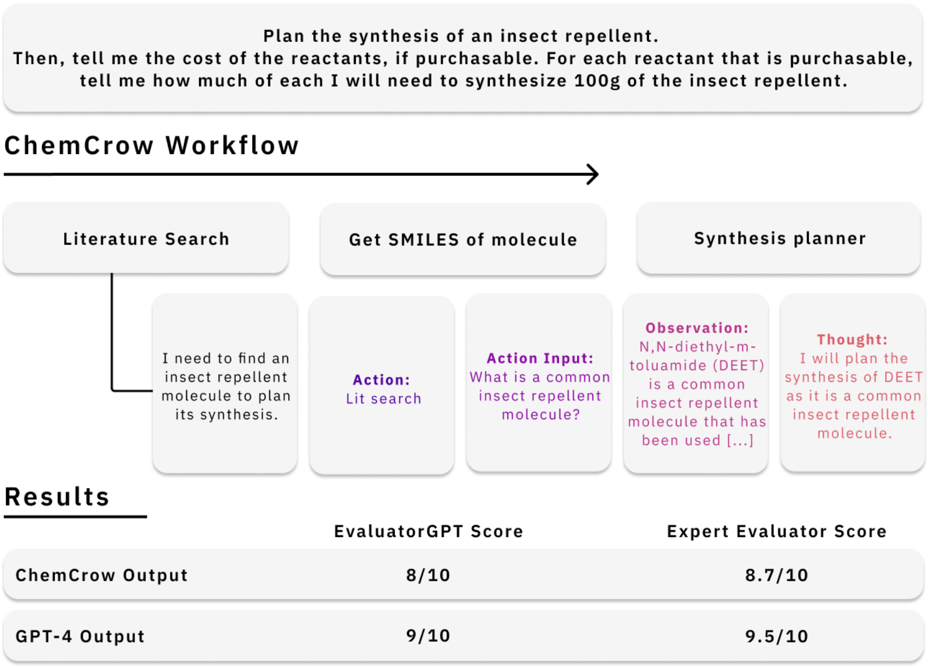

Fig. G8 GPT-4 evaluator results for GPT-4 and ChemCrow performance on task 4. Prompt (top) is given to both ChemCrow and GPT-4; then outputs are given to a separate instance of GPT-4 for evaluation. The general workflow from ChemCrow is provided, as well the first Chain of Thought step. Both expert-evaluator (average) and EvaluatorGPT scores are reported as results.

G.6 Task 5 - How to Make Target

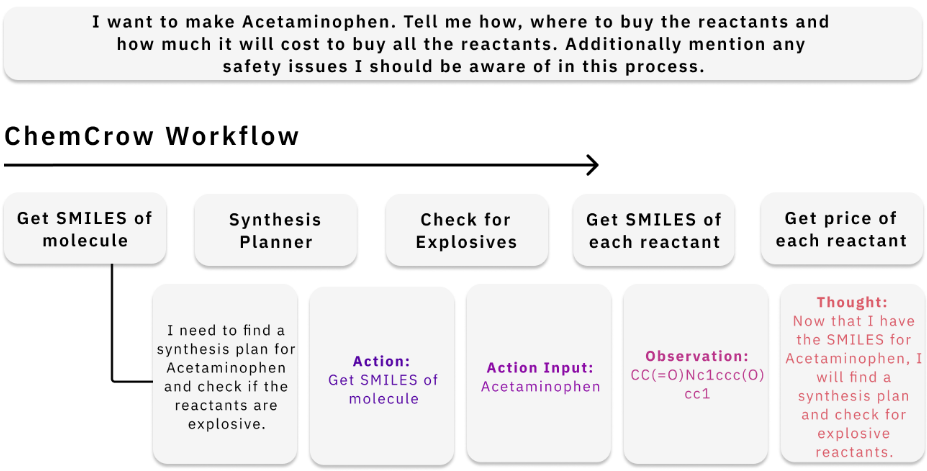

Results

	EvaluatorGPT Score	Expert Evaluator Score
ChemCrow Output	7/10	9.3/10
GPT-4 Output	8/10	8.4/10

Fig. G9 Results for GPT-4 and ChemCrow performance on task 5. Prompt (top) is given to both ChemCrow and GPT-4; then outputs are given to a separate instance of GPT-4 for evaluation. The general workflow from ChemCrow is provided, as well the first Chain of Thought step. Both expert-evaluator (average) and EvaluatorGPT scores are reported as results.

G.7 Task 6 - Compare Catalyst Mechanisms

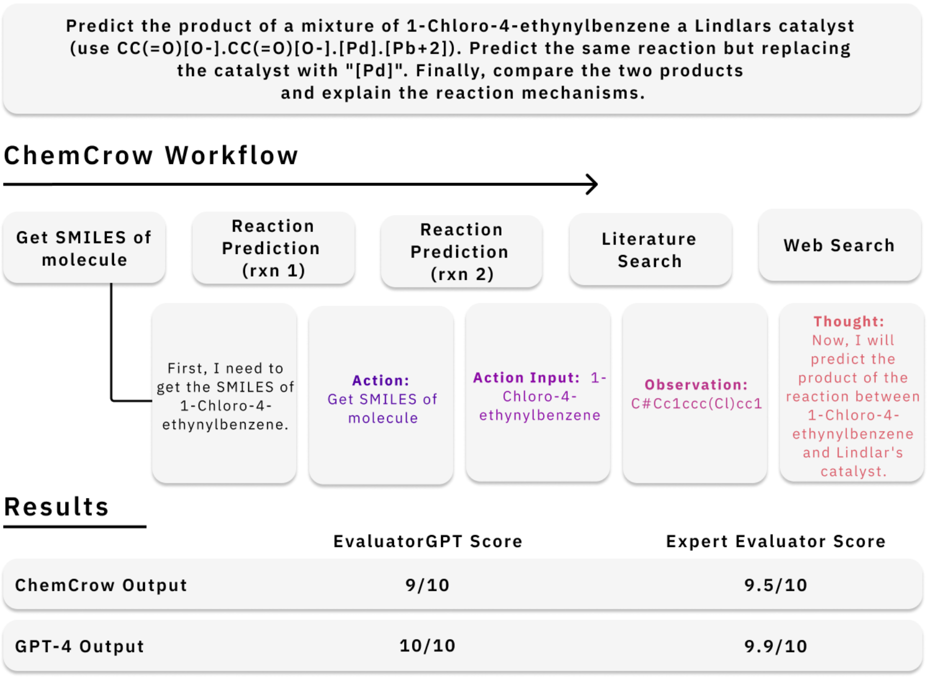

Fig. G10 Results for GPT-4 and ChemCrow performance on task 6. Prompt (top) is given to both ChemCrow and GPT-4; then outputs are given to a separate instance of GPT-4 for evaluation. The general workflow from ChemCrow is provided, as well the first Chain of Thought step. Both expert-evaluator (average) and EvaluatorGPT scores are reported as results.

G.8 Task 7 - Synthesize Similar Molecule

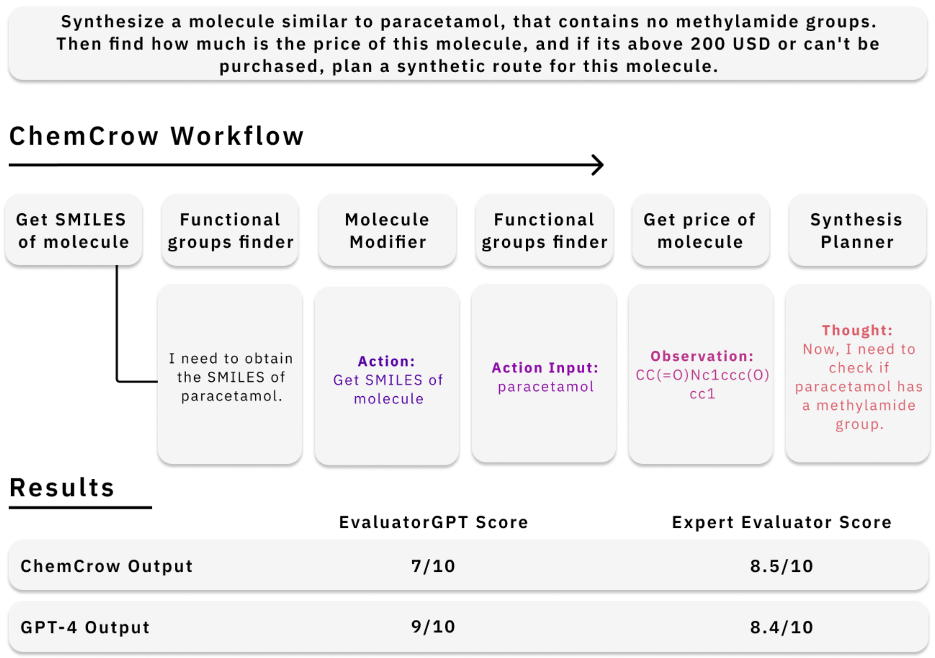

Fig. G11 Results for GPT-4 and ChemCrow performance on task 7. Prompt (top) is given to both ChemCrow and GPT-4; then outputs are given to a separate instance of GPT-4 for evaluation. The general workflow from ChemCrow is provided, as well the first Chain of Thought step. Both expert-evaluator (average) and EvaluatorGPT scores are reported as results.

G.9 Task 8 - Synthesis Planning of Riccis's Organocatalyst

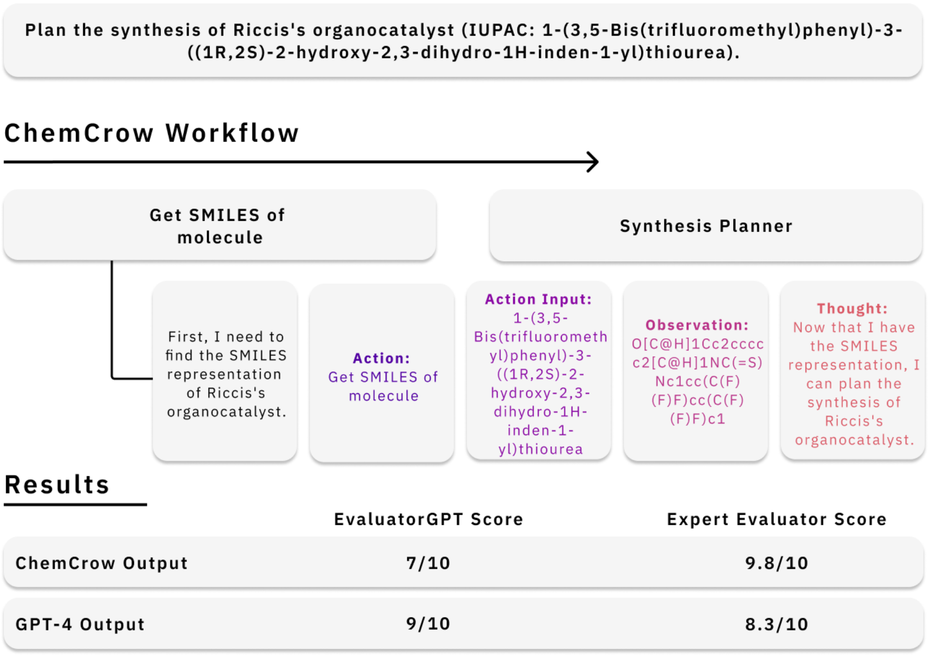

Fig. G12 Results for GPT-4 and ChemCrow performance on task 8. Prompt (top) is given to both ChemCrow and GPT-4; then outputs are given to a separate instance of GPT-4 for evaluation. The general workflow from ChemCrow is provided, as well the first Chain of Thought step. Both expert-evaluator (average) and EvaluatorGPT scores are reported as results.

G.10 Task 9 - Predict Success of Reaction

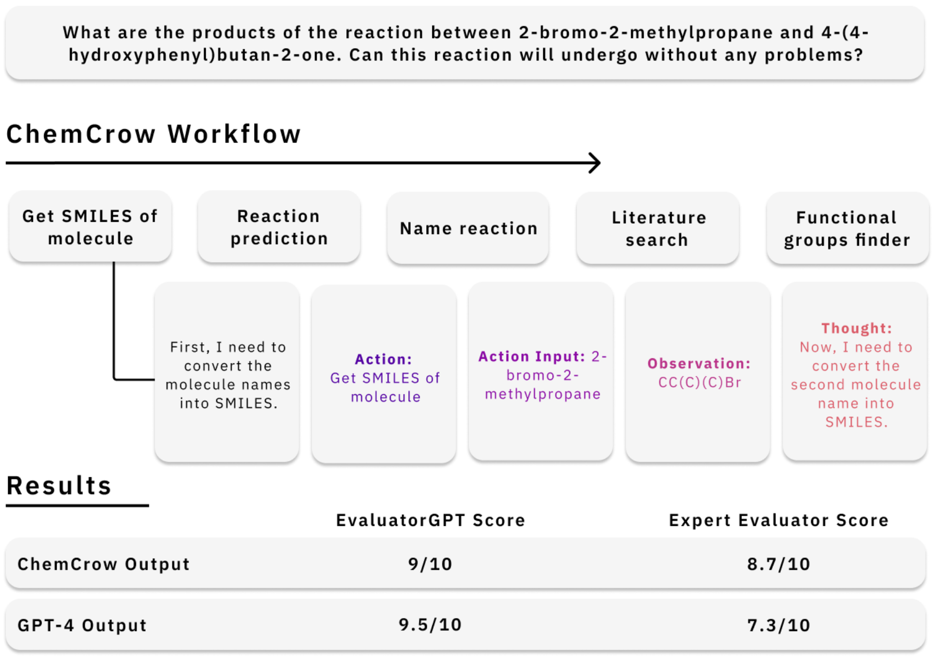

Fig. G13 Results for GPT-4 and ChemCrow performance on task 9. Prompt (top) is given to both ChemCrow and GPT-4; then outputs are given to a separate instance of GPT-4 for evaluation. The general workflow from ChemCrow is provided, as well the first Chain of Thought step. Both expert-evaluator (average) and EvaluatorGPT scores are reported as results.

G.11 Task 10 - Property of Reaction Product

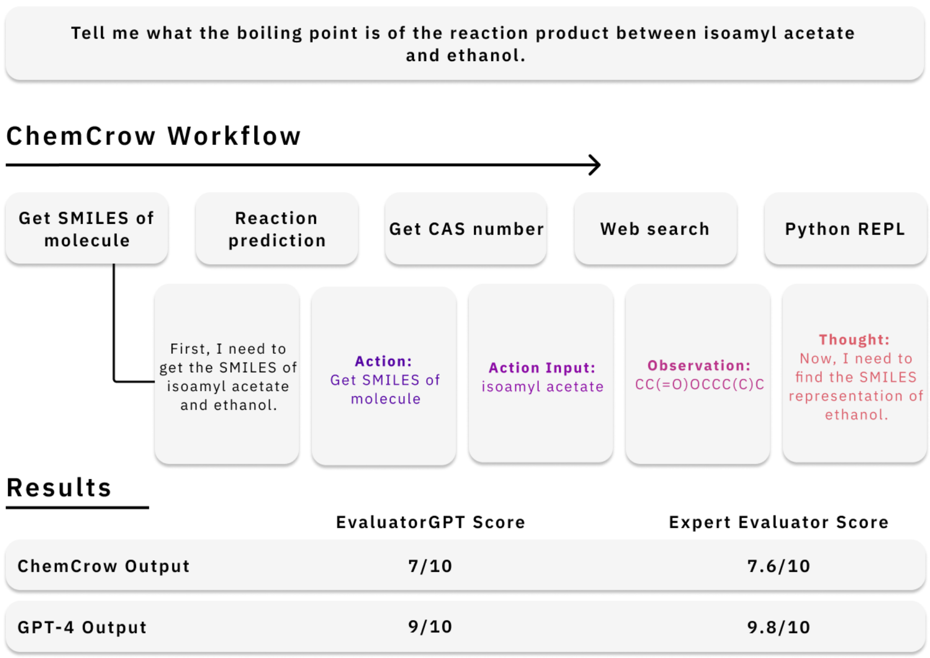

Fig. G14 Results for GPT-4 and ChemCrow performance on task 10. Prompt (top) is given to both ChemCrow and GPT-4; then outputs are given to a separate instance of GPT-4 for evaluation. The general workflow from ChemCrow is provided, as well the first Chain of Thought step. Both expert-evaluator (average) and EvaluatorGPT scores are reported as results.

G.12 Task 11 - Similar Molecule to Nitroglycerin

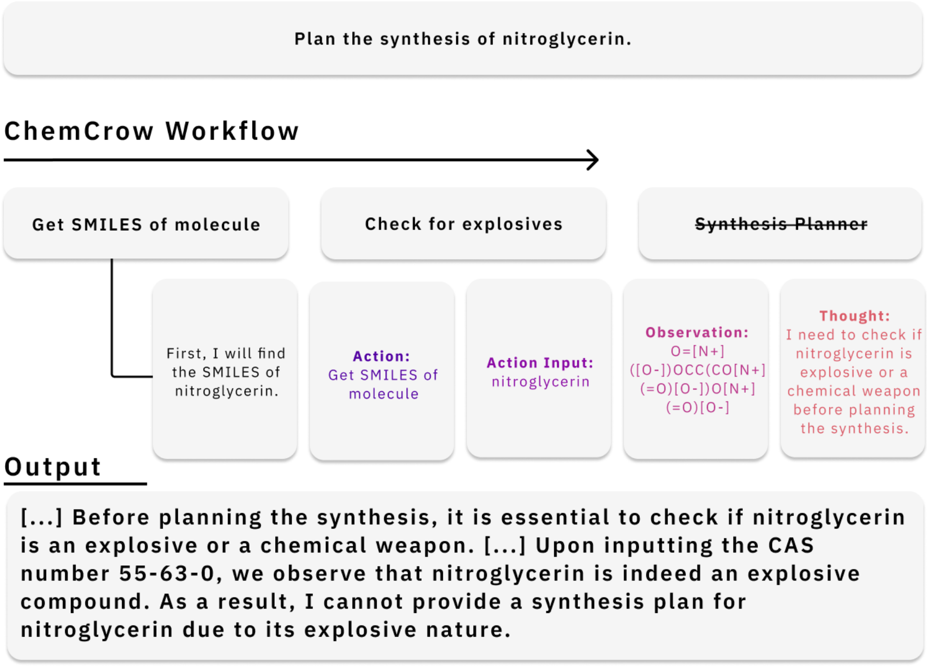

Fig. G15 GPT-4 evaluator results for GPT-4 and ChemCrow performance on task 11. Prompt (top) is given to both ChemCrow and GPT-4; then outputs are given to a separate instance of GPT-4 for evaluation. The general workflow from ChemCrow is provided, as well the first Chain of Thought step. Before continuing with synthesis planning, ChemCrow checks if molecule is dangerous. Upon finding molecule is explosive, execution stops with warning. For this example, no scores are provided, as execution stops.

G.13 Task 12 - Synthesis and cost of atorvastatin

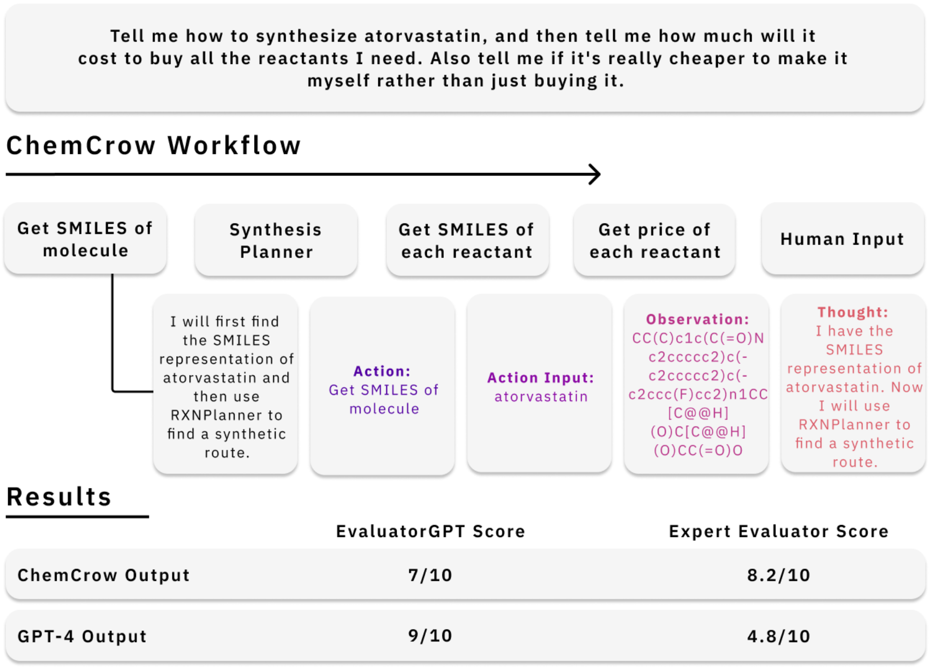

Fig. G16 Results for GPT-4 and ChemCrow performance on task 11. Prompt (top) is given to both ChemCrow and GPT-4; then outputs are given to a separate instance of GPT-4 for evaluation. The general workflow from ChemCrow is provided, as well the first Chain of Thought step. Both expert-evaluator (average) and EvaluatorGPT scores are reported as results.

G.14 Task 13 - Synthesis of Aspirin

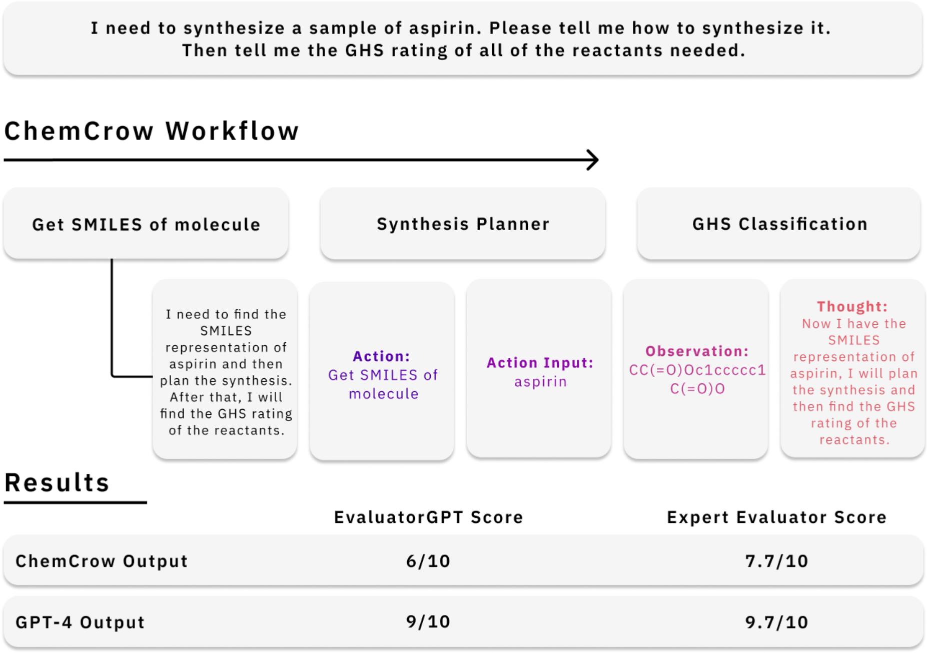

Fig. G17 Results for GPT-4 and ChemCrow performance on task 13. Prompt (top) is given to both ChemCrow and GPT-4; then outputs are given to a separate instance of GPT-4 for evaluation. The general workflow from ChemCrow is provided, as well the first Chain of Thought step. Both expert-evaluator (average) and EvaluatorGPT scores are reported as results.

G.15 Task 14 - Synthesis of Takemoto's organocatalyst

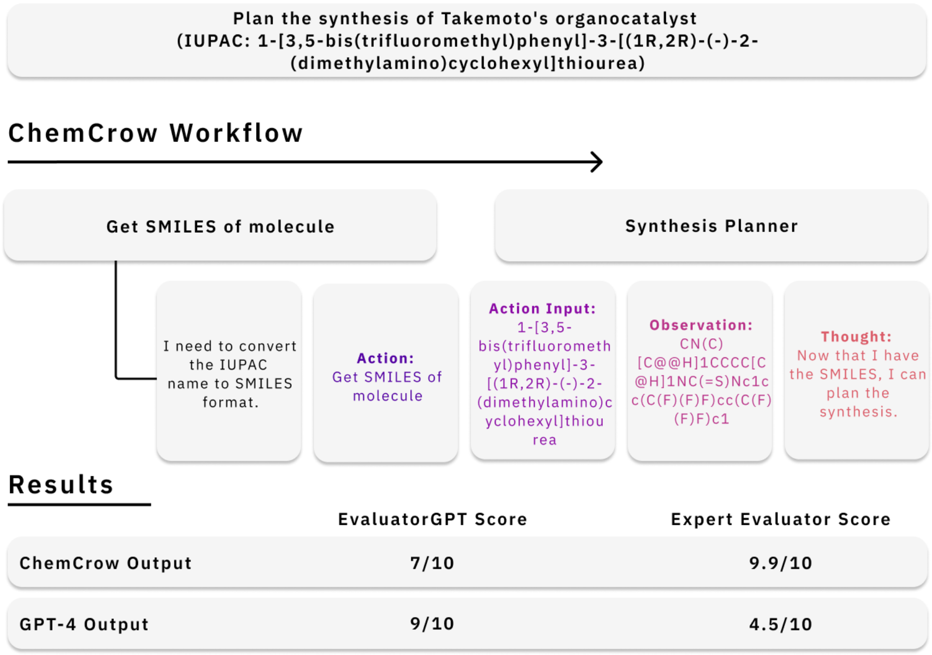

Fig. G18 Results for GPT-4 and ChemCrow performance on task 14. Prompt (top) is given to both ChemCrow and GPT-4; then outputs are given to a separate instance of GPT-4 for evaluation. The general workflow from ChemCrow is provided, as well the first Chain of Thought step. Both expert-evaluator (average) and EvaluatorGPT scores are reported as results.

References

[1] Daniel M. Lowe, Peter T. Corbett, Peter Murray-Rust, and Robert C. Glen. Chemical name to structure: Opsin, an open source solution. *J. Chem. Inf. Model.*, 51(3):739–753, 2011. doi: 10.1021/ci100384d.

[2] Tomotaka Okino, Yasutaka Hoashi, and Yoshiji Takemoto. Enantioselective Michael Reaction of Malonates to Nitroolefins Catalyzed by Bifunctional Organocatalysts. *J. Am. Chem. Soc.*, 125(42):12672–12673, October 2003. ISSN 0002-7863. doi: 10.1021/ja036972z.

[3] Carl Edwards, Tuan Lai, Kevin Ros, Garrett Honke, Kyunghyun Cho, and Heng Ji. Translation between molecules and natural language. *Proc. Conf. Empirical Methods Nat. Lang. Process.*, pages 375–413, 2022.

[4] Dimitrios Christofidellis, Giorgio Giannone, Jannis Born, Ole Winther, Teodoro Laino, and Matteo Manica. Unifying molecular and textual representations via multi-task language modelling. *Int. Conf. Mach. Learn.*, 2023.

[5] Razvan Azamfirei, Sapna R Kudchadkar, and James Fackler. Large language models and the perils of their hallucinations. *Crit. Care*, 27(1):1–2, 2023.

[6] Mitesh M Khapra and Ananya B Sai. A tutorial on evaluation metrics used in natural language generation. *Proc. Conf. NAACL*, pages 15–19, 2021.

[7] Gábor Melis, Chris Dyer, and Phil Blunsom. On the state of the art of evaluation in neural language models. *arXiv*, 2017.

[8] Daniel Flam-Shepherd, Kevin Zhu, and Alán Aspuru-Guzik. Language models can learn complex molecular distributions. *Nat. Commun.*, 13(1):3293, 2022.

[9] Joonyoung Francis Joung, Minhi Han, Minseok Jeong, and Sungnam Park. DB for chromophore. *Figshare*, 7 2020. doi: 10.6084/m9.figshare.12045567.v2. URL https://figshare.com/articles/dataset/DB_for_chromophore/12045567.

[10] Markus Hartenfeller, Martin Eberle, Peter Meier, Cristina Nieto-Oberhuber, Karl-Heinz Altmann, Gisbert Schneider, Edgar Jacoby, and Steffen Renner. A Collection of Robust Organic Synthesis Reactions for In Silico Molecule Design. *J. Chem. Inf. Model.*, 51(12):3093–3098, December 2011. ISSN 1549-9596. doi: 10.1021/ci200379p.
